# Predictors of Coordinated and Comprehensive Care Within a Medical Home for Children With Special Healthcare (CHSCN) Needs

**DOI:** 10.3389/fpubh.2018.00170

**Published:** 2018-06-07

**Authors:** Ashley Walker, John G. Peden, Morgan Emter, Gavin Colquitt

**Affiliations:** ^1^Department of Community Health Education and Behavior, Georgia Southern University, Statesboro, GA, United States; ^2^School of Human Ecology, Georgia Southern University, Statesboro, GA, United States; ^3^Department of Health Sciences and Kinesiology, Georgia Southern University, Statesboro, GA, United States

**Keywords:** disability health, children with special health care needs (CSHCN), care coordination, community-based services, public health workforce training

## Abstract

The purpose of this study was to examine predictors of coordinated and comprehensive care within a medical home among children with special health care needs (CSHCN). The latest version of the National Survey of Children with Special Health Care Needs (NS-CSHCN) employed a national random-digit-dial sample whereby US households were screened, resulting in 40,242 eligible respondents. Logistic regression analyses were performed modeling the probability of coordinated, comprehensive care in a medical home based on shared decision-making and other factors. A total of 29,845 cases were selected for inclusion in the model. Of these, 17,390 cases (58.3%) met the criteria for coordinated, comprehensive care in a medical home. Access to a community-based service systems had the greatest positive impact on coordinated, comprehensive care in a medical home. Adequate insurance coverage and being White/Caucasian were also positively associated with the dependent variable. Shared decision-making was reported by 72% of respondents and had a negative, but relatively negligible impact on coordinated, comprehensive care in a medical home. Increasing age, non-traditional family structures, urban residence, and public insurance were more influential, and negatively impacted the dependent variable. Providers and their respective organizations should seek to expand and improve health and support services at the community level.

In the late 1990s, the definition for disability was re-evaluated to expand its meaning to facilitate a shift in care options. A national agenda was set to focus efforts on providing children with special health care needs (CSHCN) and their families with a more comprehensive approach to services including a move to community-based and family-centered care (1). This continues today, and is reflected in Health People 2020 (HP2020), as a key outcome is care for CSHCN that is coordinated and focused on the family (2). However, HP2020 also states that access to adequate and affordable health care is an important factor in one's quality of life (2). People with disabilities face many barriers to accessing care that is adequate, accessible, coordinated, and family centered (3). Individuals living with disabilities, compared to those without, are more negatively impacted by a lack of access to health care services (4). Previously, parents and caregivers cited access to care as one of the primary factors impeding the overall health and quality of life of their family member living with a disability (5). Access to care is mediated by many barriers. Approximately half of individuals with disabilities cannot afford health care (3). Families have noted that access to many health care services is hindered by cost (6). Many services available to families are not covered by insurance plans, which leaves the burden of cost on family members or requires families to attempt to provide some services at home (5). The American Academy of Pediatrics (7) recommends care coordination as one strategy to alleviate challenges experienced by families caring for CSHCN.

Care coordination is defined by the American Academy of Pediatrics as “a process that links CSHCN and their families with appropriate services and resources in a coordinated effort to achieve good health” (7). Care coordination is important because CSHCN receive services from a variety health care and service providers. Needs are varied and unique, which requires a multifaceted approach. Furthermore, care coordination requires knowledge of a diverse set of medical and social services, communication with professionals, and close monitoring (8). Adequate and appropriate care coordination requires a well-developed health care plan between the families and health care provider (9). Care coordination promotes functionality and decreases the likelihood a CSHCN experiences an unmet health service need (10).

Family decision-making and adequate health insurance are listed as quality indicators for a higher quality service system within a community-oriented approach (11). These indicators can increase ease of service utilization and decrease dependence on emergency-based health services (12). Additionally, reduced access is associated with poorer health status. Children with special healthcare needs are less likely to participate in community and school activities if presenting with poorer health status and experiencing more functional limitations (13). It has been recommended that health care and community providers focus on incorporating family centered, coordinated care to improve a child's ability to be an active participant in community activities. When care coordination is lacking, family involvement in health care decisions is limited. The child's needs and the needs of the family are often left unmet. Because of the disparities which exist among CSHCN, an ecological (community-based) approach to care is recommended (14).

Children with special healthcare needs often lack proper basic care due to inadequate insurance coverage (15). When coverage is improved, CSHCN are still faced with barriers to access services such as lack of guidance, fewer service providers, and higher costs for specialized services (16). However, increasing access to additional services in the community can improve the adequacy of insurance coverage and lessen the overall impact of caring for a CSHCN on the family (17).

The Maternal and Child Health Bureau (MCHB) has consolidated these important aspects of care for CSHCN into six core outcomes that reflect both potential barriers and components of effective care: shared decision-making, coordinated and comprehensive care in a medical home, adequate insurance, early and continuous screening, ease of use of community-based services, and transition services (18). Of these six outcomes, CSHCN receiving care in a medical home is the most important in the receipt of quality care. The American Academy of Pediatrics defines a medical home as “a course of ongoing, comprehensive, coordinated, family-centered care in the child's community” (19). In the MCHB definition, coordinated and comprehensive care within a medical home has five components: usual source of care, personal doctor or nurse, care coordination, family-centered care, and getting needed referrals (18). The key difference in these definitions is the context of the community. Access to community-based services is an essential component of improving care coordination and improving the overall health care of individuals with disabilities (3).

Due to the importance of coordinated and comprehensive care within a medical home among CSHCN, the purpose of this study was to examine predictors of this outcome. We hypothesize that access to community-based services and shared decision-making among providers and families will significantly improve the likelihood that families caring for CSHCN received coordinated and comprehensive care within a medical home. However, other factors such as insurance coverage, family financial burden, hours spent caring for the child, and parental education may mediate this relationship. Therefore, we used logistic regression to examine the relationship between community-based care and care coordination while accounting for these potential covariates.

## Methods

### Data collection

Data from the most recent version of the National Survey of Children with Special Health Care Needs (NS-CSHCN; 2009–2011) was used in this study. The NS-CSHCN has been administered three times since 2001. The 2009–2011 version was the last iteration as the current survey will be shortened and folded into the National Survey on Children's Health (NSCH) (20). A national telephone survey screened 372,698 children living in 196,159 homes using the CSHCN Screener–a validated instrument that identifies children who meet the federal MCHB health-consequences-based special health care needs definition (21). Data collection was conducted by the National Center for Health Statistics (NCHS) with support from the U.S. Department of Health and Human Services, MCHB. Parents of children aged 0–17 who affirmed the presence of one or more CSHCN indicators, and whose children experienced health consequences that were expected to last at least 12 months were included in the study. A total of 59,941 CSHCN were identified. Subsequent interviews were conducted with the parents of 40,242 CSHCN. State participation ranged from 751 to 878, and data were weighted based on state population estimates (22).

### Measurement and analysis

The NS-CSHCN included questions that addressed health status, insurance coverage, access to health care services, and quality of care (e.g., family centeredness, shared decision-making, coordinated care). The MCHB identified six core outcomes for the community-based system of services required by Title V of the Maternal and Child Health Services Block Grant program and these core outcomes are reiterated in HP 2020, which included: shared decision-making, coordinated care, adequate insurance, early and continuous screening, ease of use of community-based services, and transition services (18). National Chartbook Indicators included 15 individual characteristics specific to the health of the child and receipt of individual services (23). The MCHB Core Outcomes, National Chartbook Indicators, and Stratifiers were used to model the likelihood of MCHB Core Outcome #2 (CSHCN's Receiving Ongoing, Coordinated and Comprehensive Care within a Medical Home) using binary logistic regression. Of the 40,242 total respondents, there were 29,845 complete cases with responses to MCHB Core Outcomes, National Chartbook Indicators, and Stratifiers. The coordinated, comprehensive care in a medical home variable was constructed from a composite score on five sub-components: Personal Doctor or Nurse (PDN); Usual Source for Sick and Well Care; Family Centered Care; No Problem Getting Needed Referrals; and Effective Care Coordination. All measures were dichotomous (see Table 1). CSHCN were classified as (1) Receiving Coordinated, Comprehensive Care in a Medical Home if all five sub-components were present, or (0) Not Receiving Coordinated, Comprehensive Care in a Medical Home if any of the sub-component scores were absent.

**Table 1:** Maternal and child health bureau core outcome children with special health care needs receiving ongoing, coordinated and comprehensive care within a medical home.

**Outcome/indicator**	**Components**	**Response choices**	**Criteria**
Personal doctor or nurse	a) Health professional who knows the child well and is familiar with health history	1) Yes, one 2) Yes, more than one 3) No	Answered “yes, one” or “yes, more than one”
Usual source of care	a) Has a usual source for sick care b) Has a usual source for preventative care	1) Yes 2) There is no place 3) There is more than one place	Answered “yes” or “there is more than one place” to both components
Family-centered care	a) Provider spent enough time with the child b) Provider listened carefully c) Provider was sensitive to health concerns d) Provider gave needed information e) Provider made family feel like a partner	1) Never 2) Sometimes 3) Usually 4) Always	Answered “usually” or “always” to all “components
Getting needed referrals	a) Did the child need a referral to see a doctor or receive services	1) Yes 2) No	Answered “yes” and “not a problem”
	b) Getting referrals was a problem	1) Big problem 2) Small problem 3) Not a problem	
Effective care coordination	a) Family usually or always gets sufficient help coordinating care if needed b) Doctor communicated with specialized therapist(s) if needed c) Family is very satisfied with doctors communication with each other d) Family is very satisfied with doctors communication with other programs	1) Yes 2) No	Considered “met” if usually received help when needed, were “very satisfied” with communication among providers (when needed), and communication between providers and other programs (when needed)

Covariates (see Table 2) included MCHB Core Outcome #1 (Shared Decision-Making for Child's Optimal Health), #5 (Community-Based Service Systems Organized for Ease of Use), and National Chartbook Indicators #3 (Inconsistently Insured), #5 (Adequacy of Current Insurance Coverage), #12 (Out-of-Pocket Expenses), #13 (Family Financial Burden), #14 (Hours per Week Providing Care), and #15 (Impact on Family Work Life).

**Table 2:** Maternal and child health bureau and national chartbook indicators composite variables.

**Outcome/Indicator**	**Components**	**Response choices**	**Criteria**
Shared decision-making for child's optimal health	a) Providers discussed range of treatment options b) Providers encouraged caregivers to raise concerns c) Providers made it easy to ask questions d) Providers considered and respected caregiver's thoughts regarding treatment options	1) Never 2) Sometimes 3) Usually 4) Always	Answered “usually” or “always” to all components
Community-based service systems organized for ease of use	a) Not eligible for services b) Services not available in your area c) Waiting lists or other problems getting appointments; d) Issues related to cost e) Trouble getting the information you needed f) Any other difficulties not mentioned	1) Yes 2) No	Answer of “yes” to experiencing any of the components listed considered difficulty accessing community-based services
Inconsistently insured	a) The child was not insured the entire previous 12 months b) The child was not insured at some point during the past 12 months	1) Yes 2) No	Answer of “yes” to either component considered inconsistent insurance coverage
Adequacy of insurance coverage	a) Health insurance offer benefits or cover services that meet his/her needs b) Coverage of all costs are reasonable c) Health insurance allows him/her to see the health care providers he/she needs.	1) Never 2) Sometimes 3) Usually 4) Always	Insurance coverage was deemed “inadequate” if respondents answered never or sometimes to any component
Out of pocket expenses	a) Less than $1,000 b) More than $1,000	1) Less than $250 2) $250–$500 3) $501–$1,000 4) More than $1,000	High out of pocket expenses = >$1,000
Family financial burden	Family experienced financial problems due to child's health needs	1) Yes 2) No	Answer of “yes” to considered family financial burden
Hours per week providing care	Summed total of the number of hours family members spend per week providing health care at home and coordinated care for their child	1) Less than 1 h 2) 1–4 h per week 3) 5–10 h per week 4) 11 or more h per week	Categorical variable, 11 or more hours considered “high”
Impact on family work life	a) Family members stopped working because of the child's health condition b) Family members cut down on hours worked because of the child's health condition	1) Yes 2) No	Answer of “yes” to either of the questions categorized as “negatively affected”

Stratifiers included: Age, Sex, Race, Family Structure, Insurance Type, Parental Level of Education, and Metropolitan Statistical Area. Descriptive statistics were calculated prior to modeling the impact of the covariates on coordinated, comprehensive care in a medical home. Logistic regression was used for subsequent analyses due to the dichotomous dependent variable. All analyses were conducted in SPSS Version 23.

## Results

### Descriptive results

A total of 29,845 cases were selected for inclusion in the model. Of these, 17,390 cases (58.3%) met all five criteria for coordinated, comprehensive care in a medical home. Shared decision-making was reported by 72% of respondents. Access to community-based service systems organized for ease of use was reported by 62% of respondents. Over 92% of respondents indicated that they were insured for the entire year, but approximately 36% stated that their current insurance coverage was inadequate. Out of pocket medical expenses ranged from less than $250 per year (36%) to over $1,000 per year (29.5%). CSHCN whose families experienced financial problems due to a child's health needs represented 24% of cases. Hours per week providing or coordinating health care for a child with special healthcare needs ranged from less than 1 h (38%) to more than 10 h per week (13%). A family member had to cut back on work hours or stop working entirely in approximately 27.5% of cases (see Table 3).

**Table 3:** Frequencies for variables included in logistic regression model.

**Variable**	***n***	**%**
Coordinated care-met	17,390	58.3
Coordinated care-not met	12,455	41.7
Shared-not met	8,197	27.5
Shared-met	21,477	72.0
Shared-don't know	171	0.60
Access-not met	11,160	37.4
Access-met	18,501	62.0
Access-don't know	184	0.60
Insured entire year	27,571	92.7
Inconsistently insured	2,179	7.3
Adequate insurance	18,600	64.5
Inadequate insurance	10,223	35.5
OPE < $250	10,719	36.4
OPE $250–$500	6,118	20.8
OPE $501–$1,000	3,923	13.3
OPE > $1,000	8,676	29.5
No financial problems	22,475	75.9
Financial problems	7,153	24.1
< 1 h Providing Care	10,862	37.8
1–4 h Providing Care	11,102	38.6
5–10 h providing care	3,022	10.5
≥11 h providing care	3,775	13.1
Employment not affected	21,495	72.5
Cut back or stopped working	8,138	27.5
Age 0–5	5,580	18.7
Age 6–11	11,775	39.5
Age 12–17	29,793	41.8
Male	17,980	60.3
Female	11,813	39.7
Hispanic	3,356	11.2
White, non-hispanic	20,927	70.1
Black, non-hispanic	2,794	9.4
Other, non-hispanic	2,768	9.3
Biological or adopted parent	18,368	62.4
2 Parent Stepfamily	2,730	9.3
Mother only	5,897	20.0
Other family structure	2,440	8.3
Private insurance	16,924	56.4
Public insurance	8,880	29.5
Public and private insurance	2,427	8.1
Uninsured	723	2.4
< High school	1,580	5.3
High school	4,346	14.6
More than HS	23,919	80.1
Outside of MSRA	4,285	21.0
Within MSRA	16,081	79.0

Ages of the CSHCN population ranged from 0 to 5 years (19%) to 12–17 years (42%). Males (60%) composed a majority of the CSHCN population. White, non-Hispanics composed 70% of cases, followed by Hispanics (11%), Black, non-Hispanics (9%), and other non-Hispanics (9%). Approximately 62% of CSHCN were raised in a biological or adopted parent household. Single mother households represented 20% of cases, followed by two parent stepfamily households (9%), and other family structures (8%). A majority of respondents (56%) had private insurance. Public insurance was reported by 29.5% of respondents, with 8% indicating that they held both public and private insurance. Only 2.4% stated that they were currently uninsured. The highest education level attained by parents of CSHCN ranged from less than high school (5%) to more than high school (80%). Approximately 79% of respondents indicated that they resided within a metropolitan statistical area (MSA). Those residing outside of an MSA accounted for 21% of the cases.

### Logistic regression results

The covariate model resulted in a significant improvement over the constant-only model, $X_{\left(28,\;n=29,845\right)}^{2}$ = 4757.45, *p* = 0.000. The Nagelkerke *R*^2^ was 0.315 and the Hosmer and Lemeshow Test supported an acceptable fit for the data (*p* = 0.468). The correct prediction rate increased from 58.7 to 73.3%. Shared Decision-Making for Child's Optimal Health (Outcome #1), Community-Based Service Systems Organized for Ease of Use (Outcome #5), Adequacy of Current Insurance Coverage (Indicator #5), Out-of-Pocket Expenses (Indicator #12), Family Financial Burden (Indicator #13), Hours per Week Providing Care (Indicator #14), and Impact on Family Work Life (Indicator #15) were significant predictors of coordinated, comprehensive care in a medical home. Age, Race, Family Structure, Insurance Type, Parental Education Level, and Metropolitan Statistical Area were also significant predictors of coordinated, comprehensive care in a medical home.

Odds ratios indicated that access to community-based service systems had the greatest influence on the dependent variable [Exp(B) = 2.92]. Those who had adequate insurance [Exp(B) = 1.45] and who were white (as opposed to Hispanic) were also more likely to receive coordinated, comprehensive care in a medical home [Exp(B) = 1.18]. Those who participated in shared decision-making were slightly less likely to receive coordinated, comprehensive care in a medical home [Exp(B) = 0.338]. Out-of-pocket expenses, family financial burden, more hours per week providing care, and greater impact on family work life further decreased the likelihood of coordinated, comprehensive care in a medical home. Increasing age, non-traditional family structures, public insurance, higher education levels, and residences within metropolitan statistical areas also had negative influences on coordinated, comprehensive care in a medical home (see Table 4).

**Table 4:** Determinants of Coordinated, Comprehensive Care in a Medical Home among Families of CSHCN.

**Variable**	**B**	**S.E**.	**df**	Exp(B)^b^^*^
Shared-not met^a^			2	
Shared-met	−1.084	0.254		0.338^*^
Shared-don't know	0.371	0.252		1.449
Access-not met^a^			2	
Access-met	1.073	0.038		2.923^*^
Access-don't know	−0.133	0.226		0.875
Insured entire year^a^				
Inconsistently insured	−0.061	0.082	1	0.941
Adequate Insurance^a^				
Inadequate Insurance	0.369	0.039	1	1.446^*^
OPE < $250^a^			3	
OPE $250–$500	−0.094	0.054		0.910
OPE $501–$1,000	−0.258	0.063		0.772^*^
OPE > $1,000	−0.307	0.057		0.736^*^
No financial problems^a^				
Financial problems	−0.320	0.047	1	0.726^*^
< 1 h Providing care^a^			3	
1–4 h Providing care	−0.351	0.042		0.704^*^
5–10 h Providing care	−0.403	0.064		0.668^*^
≥11 h providing care	−0.415	0.062		0.660^*^
Employment not affected^a^				
Cut back or stopped working	−0.364	0.043	1	0.695^*^
Age 0–5^a^			2	
Age 6–11	−0.101	0.049		0.904^*^
Age 12–17	−0.103	0.050		0.902^*^
Male^a^				
Female	0.029	0.036	1	1.029
Hispanic^a^			3	
White, Non-hispanic	0.169	0.057		1.184^*^
Black, Non-hispanic	0.113	0.073		1.119
Other, Non-hispanic	0.095	0.082		1.100
Biological or adopted parent^a^			3	
2 parent stepfamily	0.005	0.064		1.005
Mother only	−0.121	0.050		0.886^*^
Other family structure	−0.149	0.069		0.861^*^
Private insurance^a^			2	
Public insurance	−0.192	0.055		0.825^*^
Public and private insurance	−0.133	0.070		0.876
< High school^a^			2	
High school	−0.138	0.087		0.871
More than HS	−0.264	0.081		0.768^*^
Outside of MSA^a^				
Within MSA	−0.136	0.044	1	0.873^*^

a Baseline Category.

b Values less than 1 are less likely when compared to the baseline category. Values greater than one are more likely.

* Significant at α ≤ 0.05

## Discussion

The purpose of this study was to examine the impact of access to community-based care and shared decision-making on care coordination. To examine this relationship, we used data from the most recent version of the NS-CSHCN. Among the sample, approximately 37% of families had difficulty accessing community-based services, approximately 41% did not receive coordinated, comprehensive care in a medical home, approximately 35% did not have adequate insurance, and approximately 27% did not participate in shared decision-making. These represent high percentages of families with CSHCN not meeting key MCHB outcomes.

The results of the analysis indicate community-based care had the greatest influence on coordinated, comprehensive care in a medical home even compared to insurance adequacy. Coordinated care is a positive practice to improve well-being; however, as access to services is limited, adequate care coordination is difficult to attain (24). Only approximately 58% of families reported receiving care that was effectively coordinated. Additionally, access to community-based services was the most statistically significant mediator of care coordination. Even though many communities may lack the capacity to provide these services, there are opportunities to enhance capacity through collaboration across certain sectors. The World Health Organization (3) recommends that areas with limited resources that providers seek partnerships with schools, family, friends, and under-utilized community organizations as a starting point to identifying alternatives for support services. Families caring for CSHCN face the same realities as families with a typically developing child, but unique challenges may impede the availability of community-based services. The medical and service community must adapt care delivery to meet the needs of these families. This requires a more interactive approach between caregivers and service providers and the implementation of a more interdisciplinary approach to care (25).

Families who met the criteria for shared decision-making were slightly less likely to meet the criteria for care coordination. A closer look at the nature of the questions for shared decision-making and care coordination reveals that, while both outcomes focus on communication, the dynamics in each outcome are very different. Shared decision-making focuses on communication between the provider and the caregiver, in areas such as discussing treatment options, respecting caregiver choices, encouraging caregivers to ask questions, and respecting caregivers' choices. Care coordination involves communication among providers and the coordination of the larger health system around a primary care provider. For example, care coordination focused on communication between specialist providers, provider communication with each other, and provider communication with other programs. This could be due to a potential disconnect between the CSHCN's primary care provider who is seen on a regular basis and other specialists. Pediatric and adult or specialist providers have to be navigated differently by families with CSHCN and many families find it difficult to transition from pediatric to adult care (26). This relationship is further supported by our findings related to community-based services, whereby families were much more likely [Exp(B) = 2.92] to receive coordinated, comprehensive care in a medical home when they had access to the services they needed.

Families who have adequate care coordination are more likely to report having family centered care, being involved shared decision-making processes, and are less likely to experience issues related to receiving referrals to specialty care (27). Previously, families of CSHCN reported higher rates of satisfaction when care was family-centered (28). Families have identified a lack of coordinated care as a source of family frustration and unmet medical need (24, 27). A lack of communication among providers may also add to the difficulty and frustration experienced by families when receiving services. This lack of communication may be the result of a lack of preparation among providers. Currently, very few public health schools and programs offer disability specific training (29). Physicians have also reported frustration and lack of confidence when treating individuals with disabilities, primarily due to a lack of education and training (30).

Furthermore, white, non-Hispanic families and families with adequate insurance were more likely to receive coordinated, comprehensive care in a medical home than Hispanic families or those with inadequate health insurance coverage. Our findings support previous research by Rosen-Reynoso et al. (12), which identified disparities in access and ease of use across services. Rosen-Reynoso et al. (12) found more barriers to access to care among Hispanic families and those living in poverty. Ease of use is a complex issue and our findings further validate that adequate insurance coverage is one of many facets that should be considered. If the services are not present in the community, require a higher amount of out of pocket expenses, or are in high demand with waiting lists, accessibility is not felt by the families and their children even among those families with adequate insurance (5). Accessibility includes availability and affordability. Caregivers have previously stated that some providers for CSHCN do not bill public or private insurance plans, and require private pay (5).

The challenges families caring for a CSHCN are faced with present a global public health concern. The current World Health Organization (3) Global Disability Action Plan has three objectives: (1) remove barriers and improve access to services and programs, (2) to increase the range of support services in the community, and (3) to increase the knowledge base of international data on disability and support research in disability health. The World Health Organization (3) recommends the implementation of services at the community level as a means to eliminate health disparities experienced by those living with disability, particular among individuals in low-income and rural areas.

An unfortunate reality is that most CSHCN require variety of services to achieve and maintain an acceptable health-related quality of life, but few have access to all of the services that are needed (6). While offering community-based services (including all community stakeholders, medical care, educational resources, and community life) to those with limited access to coordinated, comprehensive care in a medical home may improve overall well-being and increase ability to participate in the community (3), this requires capacity building at the community level. The complexity of their conditions requires a systems thinking (31) approach when considering models for care coordination among families with a CSHCN. Improving care coordination for this population will require effort from providers to improve access to care within the community and the expansion of disability health systems.

## Ethics statement

This study involved secondary data analysis and was approved by the Georgia Southern University Institutional Review Board under an exempt application.

## Author contributions

AW, JP, ME, and GC contributed to this manuscript in the following ways: (1) contribution to the conception and design of the work and (2) drafting, editing, revising, and approving drafts. All authors also agree to be accountable for all aspects of the work.

### Conflict of interest statement

The authors declare that the research was conducted in the absence of any commercial or financial relationships that could be construed as a potential conflict of interest.
